# Serum Cytokines Th1, Th2, and Th17 Expression Profiling in Active Lupus Nephritis-IV: From a Southern Chinese Han Population

**DOI:** 10.1155/2016/4927530

**Published:** 2016-09-22

**Authors:** Keshav Raj Sigdel, Lihua Duan, Yin Wang, Weiping Hu, Ning Wang, Qingyi Sun, Qingyan Liu, Xiaocong Liu, Xianghua Hou, Ao Cheng, Guixiu Shi, Yanlin Zhang

**Affiliations:** ^1^Department of Nephrology, The First Affiliated Hospital of Xiamen University, Xiamen, Fujian, China; ^2^Department of Nephrology, The First Hospital of Xiamen, Fujian Medical University, Xiamen, China; ^3^Arogya Health Home, Arthritis and Rheumatic Diseases Treatment Center, Jawalakhel, Lalitpur, Nepal; ^4^Department of Rheumatology and Clinical Immunology, The First Affiliated Hospital of Xiamen University, Xiamen, Fujian, China

## Abstract

Systemic lupus erythematosus (SLE) is a chronic autoimmune disease characterized by aberrant T cell immune response. Diffuse proliferative lupus nephritis (LN-IV) is the most common, severe, and active form of lupus nephritis. In this study, we investigated the production of Th1, Th2, and Th17 cytokines in prediction of active form of LN-IV. ProcartaPlex multiplex immunoassays panels were used for detection of serum Th1, Th2, and Th17 cytokines profiling. Th1 and Th17 cytokines (IL-18, IFN-*γ*, IL-12p70, IL-6, and IL-17A) were considerably expressed in the serum of lupus nephritis IV patients in comparison to the healthy control. However, only IL18 and IL6 were higher in class IV versus class III lupus nephritis. Importantly, the ratios of Th1/Th2 (IL-18/IL-4) and Th17/Th2 (IL-17A/IL-4) were significantly elevated in LN-IV when compared with LN-III, LN-V, and healthy controls. Consistently, the serum cytokines IL-18, IL-17A, and IFN-*γ* were markedly expressed in LN-IV patient glomeruli and interstitial tissue compared to other classes of LN by IHC. ROC further suggests that IL-18 was a potential marker for LN-IV. The data from our study suggests that the early detection and quantification of these cytokines may help in prediction of active form of LN-IV.

## 1. Introduction

Systemic lupus erythematosus (SLE) is a chronic autoimmune disease characterized by loss of tolerance against nuclear autoantigens, lymphoproliferation, and multiorgan tissue inflammation. The majority of SLE patients showed severe clinical manifestations such as urine proteinuria, hematuria, urinary cast, hemolytic anemia, arthritis, and CNS involvement. The kidneys are major organs directly affected by SLE resulting in end stage renal disease (ESRD) in up to 50% of patients at onset of the SLE and over 60% of patients during the disease process [1, 2]. Lupus nephritis-IV (DPLN) is the most common, severe, and active form of nephritis; the prognosis of these classes of patient is directly proportional to the period of diagnosis and initiation of effective therapy [3, 4]. A recent large clinical study of LN has suggested that patients with LN-II and LN-III classes have favorable outcome whereas patients with LN-IV, LN-V, and LN-VI have poorer outcomes [5], which further emphasizes that early detection of active forms of LN is valuable fortitude to decrease the morbidity and mortality. Presently, renal biopsy provides information on the level of renal tissue injury, pathological activity, and severity of renal disease. However, it is an invasive procedure, with some patients reluctant to have the procedure performed.

Aberrant T cell immune response plays an essential role in the pathogenesis of LN [6]. Activated T cells can release proinflammatory cytokines that promote the activation of neutrophils and macrophages [7]. Various studies have shown that active forms of LN were strongly associated with Th1, Th2, and Th17 cells expression of peripheral cytokines and that they have strong positive correlations with SLE disease activity index (SLEDAI) [8–10]. Different kinds of T cell expression in renal tissue are homogenous to the histological picture of LN classes in human [11]. Additionally, the ratios of Th1/Th2 cytokines are significantly elevated in SLE and correlated with SLEDAI, which further suggests that an imbalance of cytokines profile mediates the inflammatory response of renal tissue during the pathogenesis process [12]. Besides patients with lupus having abnormally high levels of IL-17A along with IFN-*γ* are reported in previous studies which have shown that the increased levels of serum IL-17A act as potent inflammatory responses [12, 13]. Nonetheless, renal biopsy is universally accepted procedure and is a “gold standard method” for the definitive diagnosis of LN [14]. On the other hand, researchers are also debating on the repetitive use of renal biopsy to monitor the disease activity of LN, although it is an impracticable approach due to being an invasive procedure [15]. Thus, from a histological and morphological aspect, the essential role of Th1, Th2, and Th17 cytokines together in LN may be standby for the diagnostic prediction.

Here, our study suggests that serum proinflammatory cytokines IL-18, IL-18/IL-4, and IL-17A/IL-4 have a significant role for the development of LN-IV, and ROC further suggests IL-18 as a potential marker for LN-IV. Thus, the use of serum cytokine profiling in SLE patients may assist in the finding of histological type of LN earlier and therefore pursuing specific immunosuppressive therapy without delay, reducing the likelihood of the rapid development of ESRD.

## 2. Materials and Methods

### 2.1. Patient's Characteristics

 The study included forty-nine (45 female and 4 male) patients with newly diagnosed SLE with nephritis, who were not treated with any immunosuppressive drugs agent such as methylprednisolone (MP), cyclophosphamide (CTX), and mycophenolate (MMF). The selected patients were Han Chinese from Inpatient Department of Nephrology during the year January 2013 to November 2015. Patients fulfilled the American College of Rheumatology (ACR-1997) classification criteria for SLE with no concurrent infections being recruited in this study [16]. Patients were diagnosed by renal biopsy according to the World Health Organization (WHO-1995) classifications. They were divided into three groups, LN-III mean age (*n* = 12, 41.58 ± 5.66), LN-IV (*n* = 32, 35.28 ± 9.02), and LN-V (*n* = 5, 41.20 ± 14.34), and healthy control volunteer (*n* = 24, 37.37 ± 9.30). A healthy control individual of the same age and sex was selected from the Department of Nephrology of the First Affiliated Hospital of Xiamen University. Renal histopathology data, detailed disease history, and total SLE disease activity index (SLEDAI) were obtained from patients. SLEDAI was assessed clinically by an independent physician.

### 2.2. Ethics Statement

The study was approved by Institutional Research Board (IRB) of the First Affiliated Hospital of Xiamen University. The written informed consents were obtained from all the participants: lupus nephritis patients and volunteers. In case the participants had impaired ability to provide consent, written consents were obtained from the next-of-kin or the care giver on their behalf.

### 2.3. Sample Collections

Under aseptic techniques, early morning, fasting venous blood sample (total 5 mL) was drawn from each patient and put in a plain tube on the same day of renal biopsy. Serum was isolated by centrifugation at 1000 ×g at 20°C, but in some samples, due to the presence of high lipid content, centrifugation at 10,000 ×g for 10 minutes at 4°C was conducted. Subsequent collection of serum fraction was performed by using a standard serum separator tube. The serum was then stored at −80°C until further use.

### 2.4. Assessing Disease Activity

Disease activity is assessed by using a combination of clinical history, physical examination, organ specific functional tests, and serologic studies. Total SLEDAI score was measured on the day of serum sample collection and it was assessed clinically by an independent physician and crossmatched with our findings [17]. Total SLEDAI score was 105. Often it is divided into three categories of scoring less than 5 and 5–10, and more than 10 are considered as low, medium, and high disease activity, respectively. In our study all patients have a high SLEDAI. The mean values of SLEDAI were LN-III (13.33 ± 2.8717), LN-IV (17.22 ± 3.53), and LN-V (15.8 ± 2.49). Furthermore, renal involvement was assessed by using the renal items of the SLEDAI 2000 representing the sum of four kidney-related parameters: hematuria (>5 cells/hpf), pyuria (>5 cells/hpf), proteinuria (0.5 gm/day), and renal casts. Each SLEDAI-R item received a score of 4 [17].

### 2.5. Determination of Th1, Th2, and Th17 Expressed Cytokines and Lipocalin-2 Levels in Serum

Serum levels of IFN-*γ*, TNF-*α*, IL-2, IL-18, IL-4, IL-5, IL-6, and IL-13 were determined by using ProcartaPlex multiplex immunoassays panels, affymetrix (eBioscience, EPX110-10810-901), and Luminex 100/200. Serum levels of IL-17A and IL-10 cytokines were analyzed by ELISA kit (Human Platinum ELISA, eBioscience, an affymetrix) according to the manufacturer's instructions. Similarly, the Human Lipocalin-2/NGAL ELISA (BioVendor, RD191102200R) was used for quantitative detection of serum levels of NGAL in all groups of the patients. Each serum sample was tested in two magnetic bead wells in Human ProcataPlex panels, ELSIA for IL-17A, IL-10, and NGAL. An average result was taken and calculated as per instruction.

### 2.6. Immunohistochemical Analysis

The anti-IL-18, IL-17A, and IFN-*γ* antibody was obtained from Abcam (Cambridge, Massachusetts). After deparaffinization and rehydration, the sections were treated with 3% H_2_O_2_ followed by blocking with 10% goat serum in PBS. The sections were then stained for IL-18, IFN-*γ*, and IL-17A overnight at 4°C, followed by biotin conjugated secondary antibody. Semiquantitative evaluation was performed as previously reported. The cytokines expressions in infiltrating mononuclear cells (MNCs) of glomerulus were examined and assigned a value from 0 to 3 as follows: 0, no staining, similar to that seen in negative control samples; 1, focal staining; 2, multifocal, intense MNC staining; 3, intense staining throughout the MNCs. The glomeruli were analyzed. The score of a glomerulus was calculated with the following equation: total positive score = (% MNCs staining × 0) + (% MNCs staining × 1) + (% MNCs staining × 2) + (% MNCs staining × 3).

### 2.7. Laboratory Parameters

We have taken the important routine lab parameters to support the diagnosis of SLE with nephritis patients. These parameters which were C3, C4 (Nephelometer), CysC (Latex Particle Enhanced Immunoturbidimetric Method), 24-hour urine protein (Biuret Colorometry), ANA (Indirect Immunofluorescence, IIF), and dsDNA (*Crithidia luciliae*) were detected.

### 2.8. Statistics Analysis

SPSS statistical software, version 20.0 (SPSS, Chicago, IL), was used for data analysis. Quantitative variables were given as means ± SD, medians, and IQRs where appropriate. Kruskal-Wallis, one-way ANOVA analysis of variance test was used to compare medians for the independent samples. In case of significant overall differences, cytokine levels in LN-IV were compared with other independent groups of variables of LN and healthy control by using a two-tailed Mann–Whitney *U* test. Spearmen correlation was applied to evaluate the correlations of serum cytokines levels with SLEDAI score, routine lab parameters, and serum cytokines levels and ratios. Figures were plotted with Prism software 5.0 program. *p* value less than 0.05 was considered statistically significant. Receiver operating characteristics (ROC) curves were used to determine the best cut-off points to predict LN-IV.

## 3. Results

### 3.1. Demographic Data of Lupus Nephritis Patients and Healthy Controls

The demographics of lupus nephritis patients and healthy controls are described in the Table 1. There was no significant difference in ages and sex among the groups.

### 3.2. Serum Levels of Th1, Th2, and Th17 Cell Cytokines Expression, SLEDAI, 24 hrs Urine Protein, Cys C, C3, C4, and dsDNA

**Figure 1:**
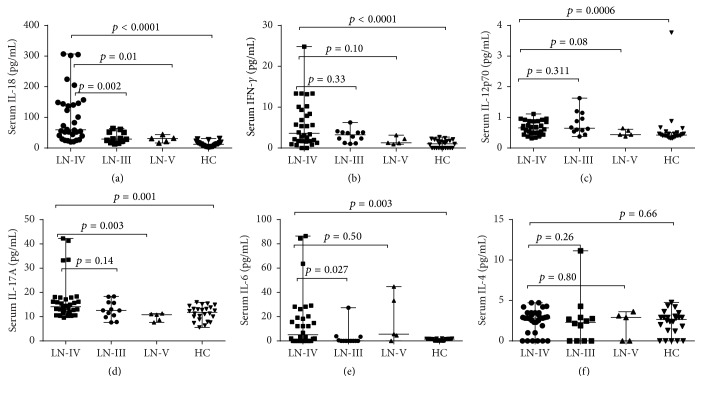
Comparison of serum levels Th1 cytokines (IFN-*γ*, IL-18, and IL-12p70), Th2 cytokine (IL-4), and Th17 cytokines (IL-17A and IL-6) in the LN classes and healthy control (HC). (a) IL-18 was significantly elevated in lupus nephritis-IV compared to III (*p* = 0.002), LN-V (0.01), and HC (*p* < 0.0001). (b) Serum IFN-*γ* level was high in LN-IV when compared with healthy control (*p* < 0.0001), whilst there are no differences among LN-III and LN-V. (c) Serum level of IL-12p70 was significantly higher in lupus nephritis-IV in comparison with HC (*p* = 0.0006), but there are no significant differences with LN-III and LN-V. (d) Serum IL-17A was elevated in LN-IV when compared to LN-V (*p* = 0.003) and healthy control (*p* = 0.001). (e) IL-6 was increased in LN-IV when compared to LN-III and healthy control. (f) IL-4 was not elevated among the groups of patients and healthy control. Mann–Whitney test was applied for each group comparison; Spearman correlation test was used to see the correlationship between two parameters. *p* value <0.05 was considered statistically significant.

Serum levels of Th1 cytokines (IFN-*γ*, IL-18, and IL-12p70) and Th17 cytokines (IL-17A and IL-6) were significantly elevated in lupus nephritis-IV when compared with healthy control. Moreover, all LN patients expressed the high level of NGAL which suggests that the renal function of patients with LN was dysfunctional (Table 2). Furthermore, serum IL-18 was compared individually in which significantly high level of IL-18 was observed in LN-IV when compared with LN-III (*p* = 0.002), LN-V (*p* = 0.01), and healthy control (*p* < 0.0001) (Figure 1(a)). In addition, only IL18 and IL6 were higher in class IV versus class III lupus nephritis. Serum levels of IL-18, IFN-*γ*, and IL-12p70 were shown to be significantly positively correlated with SLEDAI in LN-IV (*r* = 0.37; *p* = 0.03, *r* = 0.38; *p* = 0.02, and *r* = 0.59; *p* = 0.0003) respectively, but IL-18 with 24 hrs urine protein was not significantly correlated in LN-IV patients (*r* = −0.26; *p* = 0.14). However, Th2 cells cytokines (IL-4, IL-5, IL-10, and IL-13) were not elevated in our LN patient's groups when compared with healthy control. Hereby, Th1 and Th17 cytokines have a critical role in the development of LN-IV whilst Th2 cells cytokines in SLE associated nephritis may not be very specific for LN-IV.

### 3.3. Immunohistochemistry Staining and Observed in Light Microscope

**Figure 2:**
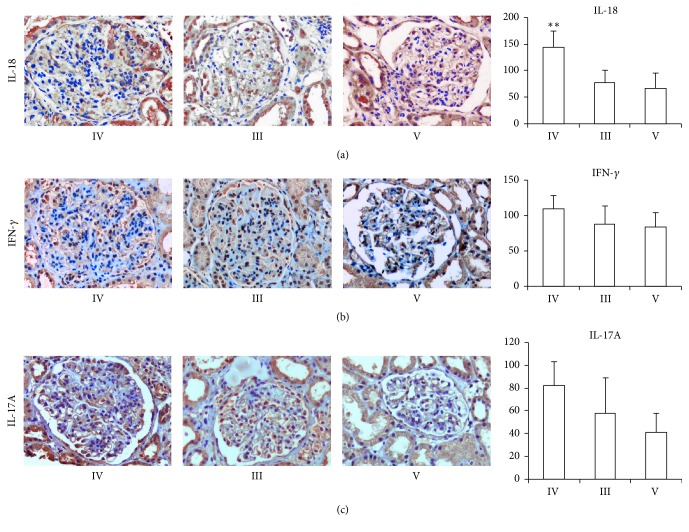
Localized expressions of IL-18, IL-17A, and IFN-*γ* in renal tissues of patients with LN-III, LN-IV, and LN-V. Expression of IL-18, IFN-*γ*, and IL-17A as shown in the level of brown color (dark to light) was detected in LN-IV patients (*n* = 5), LN-III patients (*n* = 3), and LN-V patients (*n* = 2), respectively. The cytokines expressions in infiltrating mononuclear cells (MNCs) of glomerulus were examined from 0 to 3 and glomeruli were analyzed (original magnification 200x). ^*∗∗*^ *p* value < 0.01. *p* value < 0.05 was considered statistically significant.

The depositions of the Th1 (IFN-*γ* and IL-18) and Th-17 (IL-17A) in paraffin embedded renal tissue were determined by the IHC. We have found that the considerable IL-18, IFN-*γ*, and IL-17A antibodies were deposited in renal tissue of LN-IV compared to LN-III and LN-V patients. However, only a significant difference of IL-18 expression was observed among LN-IV, LN-III, and LN-V patients (Figure 2).

### 3.4. Imbalance of Th1, Th17/Th2 (IL-18, IFN-*γ*, and IL-17A/IL-4) Cytokines in Lupus Nephritis

**Figure 3:**
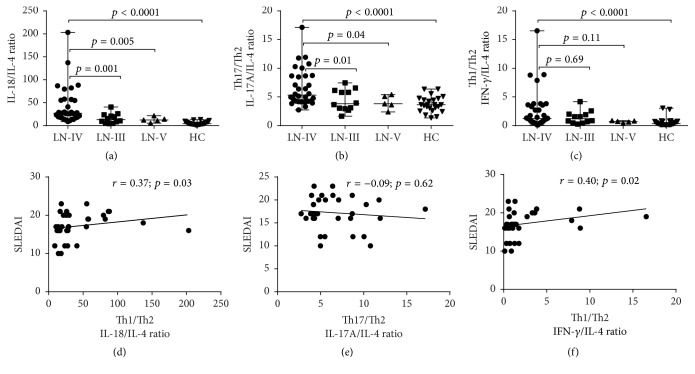
Comparison of the ratio of Th1, Th2, and Th17 cells expressed cytokines in patients with different classes of LN and healthy controls and their correlationship with SLEDAI. (a) Th1/Th2 cells cytokines IL-18/IL-4 ratio was significantly increased in LN-IV compared to other classes of LN-III (*p* = 0.001), LN-V (*p* = 0.005), and healthy control (*p* < 0.0001). (b) The Th17/Th2 cell cytokines IL-17A/IL-4 ratio was significantly higher in LN-IV when compared with LN-III (*p* = 0.04), LN-V (*p* = 0.01), and healthy control (*p* < 0.0001). (c) Similarly, Th1/Th2 cell cytokines IFN-*γ*/IL-4 ratio was significantly elevated in LN-IV compared to HC (*p* < 0.0001). IL-18/IL-4 and IFN-*γ*/IL-4 ratios were significantly correlated with SLEDAI (*r* = 0.37; *p* = 0.03 and *r* = 0.40; *p* = 0.02) in LN-IV patients (d, f). IL-17A/IL-4 negatively correlated with SLEDAI (*r* = −0.09; *p* = 0.62) but statistically was not significant (e). Mann–Whitney test was applied for group comparison and Spearman correlation test was used to see the correlationship between two parameters. *p* value < 0.05 was considered statistically significant.

Although Th2 cytokines were not changed in LN, more recent studies have been shown that Th1 and Th17 versus Th2 imbalance have a critical role in the development of LN. Thus the ratios of these cytokines were analyzed. As expected, the serum level of Th1/Th2 cytokines IL-18/IL-4 was significantly higher in LN-IV compared to other groups of lupus nephritis LN-III (*p* = 0.001), LN-V (*p* = 0.005), and healthy control (*p* < 0.0001). Similarly, the Th17/Th2 ratio was also calculated by dividing the levels of serum IL-17A by those of IL-4 (if actual observed value of IL-4 is out of range, then consider minimal sensitivity value according to protocol 1.5); IL-17A/IL-4 ratio was significantly higher in LN-IV than LN-III and LN-V. However, no significant differences were observed in LN-IV when compared with LN-III (*p* = 0.69) and LN-V (*p* = 0.11) in the ratio of IFN-*γ*/IL-4 (Figure 3). Furthermore, we found that Th1/Th2 ratios, IL-18/IL-4 and IFN-*γ*/IL-4, were significantly correlated with SLEDAI (*r* = 0.37; *p* = 0.03 and *r* = 0.40; *p* = 0.02), respectively (Figure 3). However, there was no correlation with 24 hrs urine protein (*r* = 0.05; *p* = 0.76 and *r* = 0.03; *p* = 0.83), respectively. Interestingly, we also analyzed the correlation between single cytokines, Th1/Th2 or Th17/Th2 ratio, and renal SLEDAI. As expected, we found that IFN-*γ*, IL-18, IL-17A, and Th1/Th2 ratios (IL-18/IL-4 and IFN-*γ*/IL-4) were significantly correlated with renal SLEDAI, whereas no significant correlation between IL-17A/IL-4 and renal SLEDAI was observed (Figure 5).

### 3.5. Sensitivity and Specificity of Cytokines in LN-IV Patients

**Figure 4:**
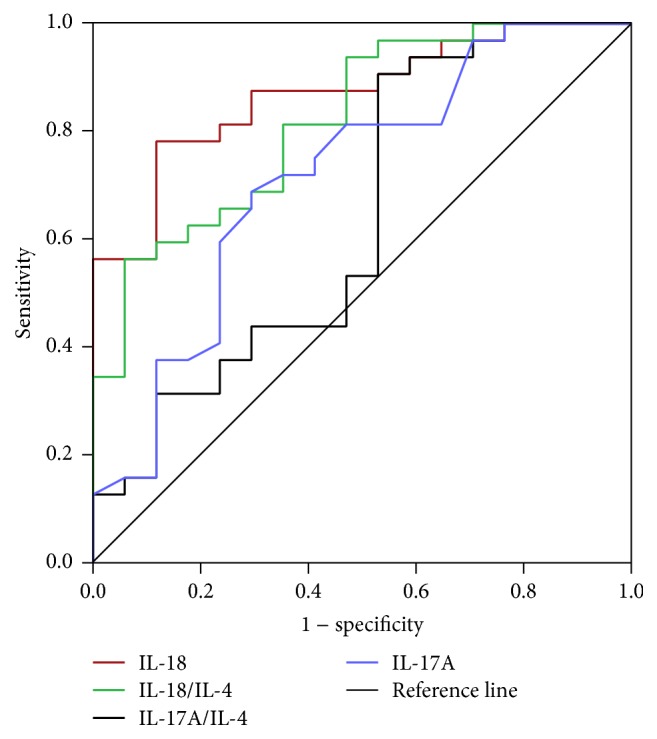
Receiver operating characteristics (ROC) curve of serum cytokines for differential diagnosis of LN-IV from LN-III and LN-V. The area under ROC curve of the serum cytokines levels IL-18, IL-17A, IL-18/IL-4, and IL-17A/IL-4 ratios was calculated and the accuracy of using these serum cytokines levels in the diagnosis of LN-IV from LN-III and LN-V was analyzed (data in Table 3).

**Figure 5:**
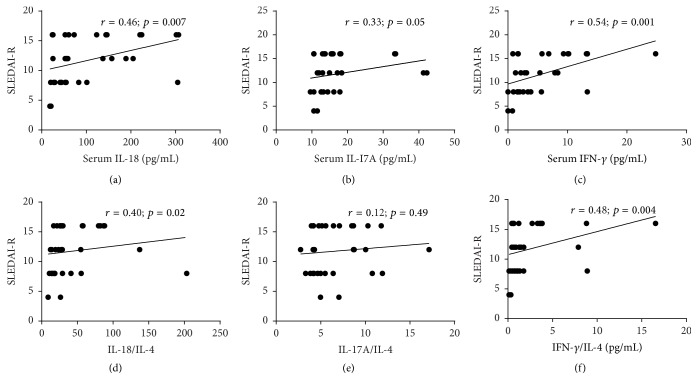
Correlation of serum levels cytokines Th1 (IL-18 and IFN-*γ*), Th17 (IL-17A), ratio of Th1/Th2 (IL-18/IL-4 and IFN-*γ*/IL-4), and Th17 (IL-17/IL-4) with renal SLEDAI. Spearman correlation test was used to analyze the correlationship between two parameters. *p* value < 0.05 was considered statistically significant.

ROC test was performed to determine the specificity and sensitivity of elevated cytokines for the diagnostic prediction of LN-IV (Table 3 and Figure 4). In short, these data showed that the crucial role of IL-18, IL-17A, and IL-18/IL-4 ratios of the cytokines during the pathogenesis of LN-IV is highly remarkable.

## 4. Discussion

Lupus nephritis has mixed morphological and histological manifestations with dissimilar clinical presentation and consequences. Early diagnosis of lupus nephritis is a challenging job for clinical practitioners without having an evidence of renal biopsy since renal biopsies cannot be performed routinely or may be delayed due to various technical issues in a hospital setting. The group of the cytokines triggered glomerular inflammation and eventually drove the irreversible renal damage. The mechanism for the pathogenesis is, however, uncertain. Interestingly, T cells could reciprocally inhibit the functions of other sets of T cells; cytokines produced by Th1 cells could negatively regulate the function of Th2 cells and vice versa [18]. The local activation of proinflammatory cytokines expressed by T cells is usually increased to the plasma level and contemplates the renal tissue pathogenesis [19]. Furthermore, cell mediated immune reactions leading to proliferative glomerulonephritis in SLE patients are predominantly mediated by Th1 cells derived cytokines [11, 20]. The balance among the Th1, Th2, and Th17 peripheral blood CD4 cells in patients with lupus nephritis is the critical determinants for histopathology picture of LN [9, 21].

Interestingly, in our study we found that Th1, Th2, and Th17 derived cytokines have a deleterious effect on the progression of different histopathological classes of LN. Since IL-18 is known as IFN-*γ* inducing cytokine factor, a novel proinflammatory cytokine endorses the polarization of the immune response towards the Th1 cells and its level was markedly elevated in serum and prominent in glomerular tissue staining in LN-IV patients compared to LN-V [8]. Consistently, our findings evokes that increased serum level of IL-18 was present in LN-IV patients compared to LN-III (*p* = 0.002), LN-V (*p* = 0.01), and healthy controls (*p* < 0.0001). A previous study also suggested that the major site of IL-18 expression in LN is the glomerulus, specifically in LN-IV which may be provided by activated glomerular infiltrating macrophages [20]. Histopathological active stage nephritic patients may have more chances to pile up these macrophages within the glomeruli and subsequent release into circulation as a defense mechanism. In such conditions, local expression of IL-18 recruited inflammatory cells from peripheral blood into the glomeruli via IL-18R [22] and polarization of the immune response towards the Th1. This may be the reason for elevation of serum IL-18 in our LN-IV patients [8]. Since a decade, the characterizations of IL-18 and the therapeutic approach to SLE are debatable despite its critical value for the development of LN [8, 20]. Recently, a mouse model study of LN has revealed that antroquinonol inhibited T cell activation/proliferation reduced the renal production of interleukin-18. It had ameliorated the proteinuria, hematuria, impairment of renal function, and development of severe renal lesions, especially cellular crescent formation, neutrophil infiltration, and T cell proliferation in the glomerulus, as well as periglomerular interstitial inflammation [23].

Subsequently, Th1 and Th2 cytokines are in counterbalance state in glomeruli of DPLN and shift towards IFN-*γ* and away from Th2 cytokines [10, 11]. However, IFN-*γ* and IL-4 antagonize the Th-17 pathway in a complex fashion. Reciprocal interaction among IFN-*γ*, IL-4 and, IL-17A occurred, with IL-17A expression characterizing an exceptional role of T helper cells to regulate the tissue inflammation [24]. Similarly, in our study we found that the ratios of cytokine Th1/Th2 and IL-18/IL-4 were profoundly elevated in LN-IV compared to LN-III (*p* = 0.001), LN-V (*p* = 0.005), and healthy control (*p* < 0.0001) and significantly correlated with SLEDAI (*r* = 0.37; *p* = 0.03). Consequently, IFN-*γ*/IL-4 also significantly elevated in LN-IV in comparison to healthy control (*p* < 0.0001). Hence, the ratios of Th1 and Th2 cytokines can reflect the cytokines homeostasis and point out the predominance of either Th1 or Th2 cells cytokines during the pathogenesis of lupus. These ratios were correlated with SLEDAI significantly [12]. Although IL-4 cytokine in the sera was not significantly elevated in our study, the ratios of Th1/Th2 cytokines were markedly increased particularly in LN-IV patients. It may be due to counterbalance characteristics of T cell subsets during inflammatory processes as T cells could reciprocally inhibit the functions of other subsets of T cells. Cytokines produced by Th1 cells could negatively regulate the function of Th2 cells and vice versa during active inflammation [18]. A previous study has also revealed that the Th1/Th2 ratio was higher in SLE patients and positively correlated with SLEDAI [12]. In contrast, a study showed that Th1/Th2 ratio was significantly correlated with 24 hrs urine protein but not with SLEDAI [8]. However, in our study high ratio of Th1/Th2 was significantly correlated with SLEDAI in LN-IV whereas it was not correlated with 24 hrs urine protein and other lab parameters. Furthermore, our study also found that renal SLEDAI-R was significantly correlated with single IL18, IFN-*γ*, IL-17A, and Th1/Th2 ratio. Consistently, previous study showed that, at the time of renal biopsy, SLEDAI-R scores were significantly higher in LN-IV, superior to other indices to predict for renal injury in children with LN [25]. The enhancement of proinflammatory reaction induced tissue injury is varied in renal tissue and exacerbation of SLEDAI correlated with active stage of LN, especially LN-IV.

Th-17 is newly identified T helper subset cell and it became a hallmark of pathogenesis in various autoimmune diseases. Recently, various studies have reported that IL-17A level in SLE is elevated when compared with healthy controls [26–28]. Localized prominent expression of IL-17A and IFN-*γ* occurred in renal tissue of patients with LN-III and LN-IV compared to LN-II by IHC staining. As a result, Th17 cells appeared to be responsible for tissue inflammation and damage [29, 30]. Different studies have shown that serum IL-17A level was significantly elevated in LN patients in comparison to SLE patient without nephritis and healthy controls, but it has no associations with clinical and laboratory parameters such as white blood cells, platelet, hematuria, dsDNA, ANA, C3, and C4 [31]. In contrast, IL-17A and IFN-*γ* were significantly amplified in SLE; mainly IL-17A acts as a powerful predictor cytokine because it was markedly correlated with disease activity and associated with more pyuria and proteinuria, whilst the serum level of IFN-*γ* was associated with more pyuria and hematuria [28]. In our study, IL-17A levels were significantly elevated in LN, especially with LN-IV compared to LN-V (*p* = 0.003) and healthy control (*p* = 0.001). Nonetheless, no significant correlation between serum IL-17A levels and SLEDAI was observed in LN patients, in keeping with similar finding from a previous study [31]. It may be due to heterogeneity of SLE with nephritis or a small sample size. Previous studies have also shown that an enhanced Th17 cell response that directly correlates with disease activity in patients with SLE suggests the important role of IL-17A in the pathogenesis of lupus. However, there was a balance mechanism between Th1 (IFN-*γ*) and Th17 (IL-17A) regulation and aberrantly production of IL-6 was present in SLE, which is linked to an increased Th17 cell response [32]. Therefore, patients with SLE had an increased frequency of CD4^+^ Th17^+^ cells compared with healthy controls, but it has altered the balance between Th17 and Th1 response in SLE. Wang et al. [33] have shown that the IL-17A expressing T cells predominantly infiltrated in glomerular and interstitial tissue. Moreover, therapeutic blockade of IL-17A may offer a therapeutic target of LN. Thus IL-17A may have undoubted role in the pathogenesis of LN. In contrast, a recent study showed that IL-17A immune response plays no major role in the pathogenesis of LN in MRL/lpr and NZB/WF-1 mice, which concludes that the IL-17A targeting therapy may not be supported [34]. With such controversy, furthermore, it was noticed that serum IL-17A cytokine is elevated in SLE patients with CNS involvement and raised Th17/Th2 ratio and it has correlated with SLEDAI [35]. It may be due to the fact that IL-17A levels are produced by several cell types including CD4^+^ Th cells, *γδ* T cells, double negative T cells, and Foxp3^+^Treg cells [36, 37]. Nevertheless, we found that serum IL-17A levels were significantly higher in LN-IV patients than LN-V and healthy controls. In accordance with this result, we have further investigated the expression of Th1 cells cytokines (IL18 and IFN-*γ*) and Th17 cells cytokines IL-17A in paraffin embedded renal biopsy tissues through IHC staining method. The IL-18, IFN-*γ*, and IL-17A were significantly deposited on renal biopsy tissue from class IV LN patients in comparison to LN-III and LN-V (Figure 2). The infiltration of Th1 and Th17 cells into glomeruli was consistent with previous studies [8, 9, 11]. Hereby, we conclude that these cytokines are crucial for the development of LN, especially LN-IV. Although IL-6 was higher in patient with LN-IV than in healthy control subjects in our study, it may be due to possible involvement of this cytokine in enhancing the Th17 cell response [32].

In our study, we found that IL-18 and IL-18/IL-4 ratios were powerful predictor (in ROC curve) of the serum cytokines and significantly correlated with total and renal SLEDAI in LN-IV. This result was supported by other studies which has reported that the cytokine production is increased in active DPLN nephritis patients [8, 12, 20]. Similarly, IL-17/IL-4 ratio also increased in LN-IV but it was not correlated with SLEDAI significantly; however, it was consistent with previous studies [28, 31].

## 5. Conclusions 

In conclusion, T cell subset plays a pivotal role in pathogenesis of LN. Particularly, in our study the serum IL-18, IL-18/IL-4, and IL-17/IL-4 were the most powerful predictor cytokines in LN-IV patients. Determination of these T cell subsets cytokine profiling in SLE patients with NGAL may assist in early prediction of LN-IV before renal biopsy and guide a clinician to initiate specific immunosuppressive therapy without delay and reduce the tendency of ESRD. Hereby, the measurement of these cytokines may immediately help to predict the active form of lupus nephritis, especially LN-IV prior to renal biopsy; if biopsy could not be readily performed. Nevertheless, until then, renal biopsy is the gold standard method to establish the definitive diagnosis of LN.

## Figures and Tables

**Table 1:** Baseline demographic and laboratory parameters.

Characteristics	Group of patients			Healthy control	*p* value
	LN-III (*n* = 12)	LN-IV (*n* = 32)	LN-V (*n* = 5)	HC (*n* = 24)	
Age years	41.58 ± 5.66	35.28 ± 9.02	41.20 ± 14.34	337.37 ± 9.30	0.190
Sex F/M	12/0	29/3	4/1	21/3	0.417
SLEDAI	13.33 ± 2.8717	17.22 ± 3.53	15.8 ± 2.49	nd	0.003 (^*∗*^0.0006, ^&^0.18, ^#^0.3)
Urine protein, g/24 hrs	1.64 ± 0.37	3.1 ± 2.89	1.96 ± 1.06	nd	0.348
C3, g/L	0.45 ± 0.23	0.40 ± 0.24	0.37 ± 0.22	nd	0.756
C4, g/L	0.10 ± 0.07	0.12 ± 0.12	0.07 ± 0.06	nd	0.736
CysC, mg/L	1.90 ± 1.43	2.09 ± 0.84	1.76 ± 0.31	nd	0.065
dsDNA, Iu/mL^*∗*^	107.96 (57.14–150.41)	130.5 (22.93–600.8)	136.4 (87.34–447.7)	nd	0.781
NGAL, ng/mL^*∗*^	89.58 (15.37–251.22)	88.07 (27.01–190.55)	59.9 (28.84–106.68)	5.43 (3.83–10.17)	<0.0001 (^*∗*^0.91, ^&^0.64, ^#^0.81)

Ages and sex differences determined by Fischer test, then SLEDAI, C3, C4, 24-hour urine protein, and cystatin-C are expressed in mean ± SD. Kruskal-Wallis test was applied for groups analysis. SLEDAI: systemic lupus erythematosus disease activity index; significant differences were found in LN-IV (*p* = 0.003) when compared with LN-III and LN-V. NGAL (neutrophil gelatinase-associated lipocalin) was significantly elevated in all classes of LN when compared with healthy control (*p* < 0.0001) (nd = not determined; ns = not significant). ^*∗*^LN-III compared with LN-IV, ^&^LN-IV compared with LN-V, and ^#^LN-III compared with LN-V. *p* value < 0.05 was considered statistically significant.

**Table 2:** Comparisons of serum Th1, Th2, and Th17 cell expressed cytokines in different classes of lupus nephritis and healthy control.

Cytokines	Group of patients			Healthy control	*p* value
	LN-III (*n* = 12)	LN-IV (*n* = 32)	LN-V (*n* = 5)	HC (*n* = 24)	
*Th1*					
IFN-*γ* (pg/mL)	2.58 (1.08–3.83)	3.60 (1.68–9.09)	1.30 (0.0–2.69)	1.04 (0.0–1.79)	<0.0001
IL-18 (pg/mL)	29.36 (19.26–51.95)	59.58 (39.87–145.77)	31.06 (19.08–38.15)	12.08 (4.89–17.63)	<0.0001
IL-12p70 (pg/mL)	0.65 (0.55–1.10)	0.66 (0.49–0.86)	0.44 (0.41–0.61)	0.42 (0.38–50)	<0.0006
TNF-*α* (pg/mL)	0.75 (0.0–1.68)	1.06 (0.0–1.68)	0.0 (0.0–1.68)	1.11 (1.31–1.87)	0.231
IL-2 (pg/mL)	2.70 (1.70–3.22)	2.70 (1.56–2.96)	2.15 (0.0–2.82)	2.42 (0.0–3.22)	0.618
*Th2*					
IL-4 (pg/mL)	2.31 (0.0–2.86)	2.82 (1.22–3.48)	2.90 (0.0–3.36)	2.66 (1.22–3.05)	0.717
IL-5 (pg/mL)	0.63 (0.0–1.54)	0.0 (0.0–1.42)	0.0 (0.0–1.54)	1.5 (0.0–1.74)	0.203
IL-10 (pg/mL)	9.66 (4.34–14.86)	8.69 (4.12–14.01)	7.13 (4.72–8.62)	4.82 (3.26–8.54)	0.168
IL-13 (pg/mL)	0.57 (0.0–1.37)	0.725 (0.02–1.79)	0.8 (0.0–1.005)	0.69 (0.0–1.07)	0.633
*Th17*					
IL-6 (pg/mL)	0.0 (0.0–2.75)	5.1 (0.0–19.96)	5.6 (2.28–38.98)	1.50 (0.0–1.74)	0.003
IL-17A (pg/mL)	12.57 (10.01–15.86)	14.09 (11.65–17.69)	10.80 (8.15–11.17)	11.80 (9.15–13.68)	0.002

Comparison of serum Th1, Th2, and Th17 cells expressed cytokines among different groups. The values are expressed in median with range. Kruskal-Wallis test was applied for groups; *p* value < 0.05 was considered statistically significant.

**Table 3:** Serum IL18, IL-18/IL-4, and IL-17A/IL-4 assays using optimal cut-off values.

Assay	Cut-off	Sensitivity	Specificity	AUC	95% CI
IL-18	52.34	0.781	0.882	0.869	0.711–0.968
IL-18/IL-4	26.06	0.563	0.941	0.820	0.700–0.940
IL-17A/IL-4	3.94	0.906	0.471	0.640	0.465–0.815
IL-17A	12.67	0.688	0.706	0.717	0.651–0.873

Performance characteristics of cytokines level in LN-IV. Receiver operating characteristic curve (shown as area under the curve [AUC]) demonstrates the predictive capability of IL-18 level, IL-17A level, IL-18/IL-4 ratio, and IL-17A/IL-4 ratio for the diagnosis of LN-IV. CI: confidence interval.

## References

[1] Bomback A. S., Appel G. B. (2010). Updates on the treatment of lupus nephritis. *Journal of the American Society of Nephrology*.

[2] Hahn B. H., McMahon M. A., Wilkinson A. (2012). American College of Rheumatology guidelines for screening, treatment, and management of lupus nephritis. *Arthritis Care & Research*.

[3] Faurschou M., Starklint H., Halberg P., Jacobsen S. (2006). Prognostic factors in lupus nephritis: diagnostic and therapeutic delay increases the risk of terminal renal failure. *Journal of Rheumatology*.

[4] Fine D. M. (2005). Pharmacological therapy of lupus nephritis. *The Journal of the American Medical Association*.

[5] Tang Y., Zhang X., Ji L. (2015). Clinicopathological and outcome analysis of adult lupus nephritis patients in China. *International Urology and Nephrology*.

[6] Mak A., Kow N. Y. (2014). The pathology of T cells in systemic lupus erythematosus. *Journal of Immunology Research*.

[7] Li Y., Fang X., Li Q. Z. (2013). Biomarker profiling for lupus nephritis. *Genomics, Proteomics & Bioinformatics*.

[8] Liu X., Bao C., Hu D. (2012). Elevated interleukin-18 and skewed Th1:Th2 immune response in lupus nephritis. *Rheumatology International*.

[9] Chen D. Y., Chen Y. M., Wen M. C., Hsieh T. Y., Hung W. T., Lan J. L. (2012). The potential role of Th17 cells and Th17-related cytokines in the pathogenesis of lupus nephritis. *Lupus*.

[10] Uhm W. S., Na K., Song G. W. (2003). Cytokine balance in kidney tissue from lupus nephritis patients. *Rheumatology*.

[11] Masutani K., Akahoshi M., Tsuruya K. (2001). Predominance of Th1 immune response in diffuse proliferative lupus nephritis. *Arthritis and Rheumatism*.

[12] Wong C. K., Ho C. Y., Li E. K., Lam C. W. K. (2000). Elevation of proinflammatory cytokine (IL-18, IL-17, IL-12) and Th2 cytokine (IL-4) concentrations in patients with systemic lupus erythematosus. *Lupus*.

[13] Doreau A., Belot A., Bastid J. (2009). Interleukin 17 acts in synergy with B cell-activating factor to influence B cell biology and the pathophysiology of systemic lupus erythematosus. *Nature Immunology*.

[14] Weening J. J., D'Agati V. D., Schwartz M. M. (2004). The classification of glomerulonephritis in systemic lupus erythematosus revisited. *Journal of the American Society of Nephrology*.

[15] Moroni G., Radice A., Giammarresi G. (2009). Are laboratory tests useful for monitoring the activity of lupus nephritis? A 6-year prospective study in a cohort of 228 patients with lupus nephritis. *Annals of the Rheumatic Diseases*.

[16] Hochberg M. C. (1997). Updating the American College of Rheumatology revised criteria for the classification of systemic lupus erythematosus. *Arthritis and Rheumatism*.

[17] Lam G. K., Petri M. (2005). Assessment of systemic lupus erythematosus. *Clinical and Experimental Rheumatology*.

[18] Coffman R. L. (2006). Origins of the T_H_1-T_H_2 model: a personal perspective. *Nature Immunology*.

[19] Schwartz N., Goilav B., Putterman C. (2014). The pathogenesis, diagnosis and treatment of lupus nephritis. *Current Opinion in Rheumatology*.

[20] Calvani N., Richards H. B., Tucci M., Pannarale G., Silvestris F. (2004). Up-regulation of IL-18 and predominance of a Th1 immune response is a hallmark of lupus nephritis. *Clinical and Experimental Immunology*.

[21] Miyake K., Akahoshi M., Nakashima H. (2011). Th subset balance in lupus nephritis. *Journal of Biomedicine & Biotechnology*.

[22] Tucci M., Quatraro C., Lombardi L., Pellegrino C., Dammacco F., Silvestris F. (2008). Glomerular accumulation of plasmacytoid dendritic cells in active lupus nephritis: role of interleukin-18. *Arthritis & Rheumatism*.

[23] Tsai P.-Y., Ka S.-M., Chang J.-M. (2012). Antroquinonol differentially modulates T cell activity and reduces interleukin-18 production, but enhances Nrf2 activation, in murine accelerated severe lupus nephritis. *Arthritis and Rheumatism*.

[24] Park H., Li Z., Yang X. O. (2005). A distinct lineage of CD4 T cells regulates tissue inflammation by producing interleukin 17. *Nature Immunology*.

[25] Mina R., Abulaban K., Klein-Gitelman M. S. (2015). Validation of the lupus nephritis clinical indices in childhood-onset systemic lupus erythematosus. *Arthritis Care & Research*.

[26] Chen X. Q., Yu Y. C., Deng H. H. (2010). Plasma IL-17A is increased in new-onset SLE patients and associated with disease activity. *Journal of Clinical Immunology*.

[27] Yang X.-Y., Wang H.-Y., Zhao X.-Y., Wang L.-J., Lv Q.-H., Wang Q.-Q. (2013). Th22, but not Th17 might be a good index to predict the tissue involvement of systemic lupus erythematosus. *Journal of Clinical Immunology*.

[28] Elewa E. A., Zakaria O., Mohamed E. I., Boghdadi G. (2014). The role of interleukins 4, 17 and interferon gamma as biomarkers in patients with Systemic Lupus Erythematosus and their correlation with disease activity. *Egyptian Rheumatologist*.

[29] Yazici M. U., Orhan D., Kale G., Besbas N., Ozen S. (2014). Studying IFN-gamma, IL-17 and FOXP3 in pediatric lupus nephritis. *Pediatric Nephrology*.

[30] Rother N., van der Vlag J. (2015). Disturbed T cell signaling and altered Th17 and regulatory T cell subsets in the pathogenesis of systemic lupus erythematosus. *Frontiers in Immunology*.

[31] Zhao X. F., Pan H. F., Yuan H. (2010). Increased serum interleukin 17 in patients with systemic lupus erythematosus. *Molecular Biology Reports*.

[32] Shah K., Lee W. W., Lee S. H. (2010). Dysregulated balance of Th17 and Th1 cells in systemic lupus erythematosus. *Arthritis Research & Therapy*.

[33] Wang Y., Ito S., Chino Y. (2010). Laser microdissection-based analysis of cytokine balance in the kidneys of patients with lupus nephritis. *Clinical & Experimental Immunology*.

[34] Schmidt T., Paust H. J., Krebs C. F. (2015). Function of the T 17/IL-17A immune response in murine lupus nephritis. *Arthritis & Rheumatology*.

[35] Vincent F. B., Northcott M., Hoi A., Mackay F., Morand E. F. (2013). Clinical associations of serum interleukin-17 in systemic lupus erythematosus. *Arthritis Research & Therapy*.

[36] Xing Q., Wang B., Su H., Cui J., Li J. (2012). Elevated Th17 cells are accompanied by FoxP3+ Treg cells decrease in patients with lupus nephritis. *Rheumatology International*.

[37] Martin J. C., Baeten D. L., Josien R. (2014). Emerging role of IL-17 and Th17 cells in systemic lupus erythematosus. *Clinical Immunology*.

